# Early Detection of Malignant Transformation in Resected WHO II Low-Grade Glioma Using Diffusion Tensor-Derived Quantitative Measures

**DOI:** 10.1371/journal.pone.0164679

**Published:** 2016-10-14

**Authors:** Martin T. Freitag, Klaus H. Maier-Hein, Francisczek Binczyk, Frederik B. Laun, Christian Weber, David Bonekamp, Rafal Tarnawski, Barbara Bobek-Billewicz, Joanna Polanska, Henryk Majchrzak, Bram Stieltjes

**Affiliations:** 1 Quantitative Imaging-based Disease Characterization, German Cancer Research Center, Heidelberg, Germany; 2 Department of Radiology, German Cancer Research Center, Heidelberg, Germany; 3 Junior Group Medical Image Computing, German Cancer Research Center, Heidelberg, Germany; 4 Silesian University of Technology, Data Mining Group, Gliwice, Poland; 5 Division of Medical Physics in Radiology, German Cancer Research Center, Heidelberg, Germany; 6 Maria Sklodowska-Curie Memorial Cancer Center and Institute of Oncology, Department of Radiology, Gliwice, Poland; 7 Department of Neurosurgery, Medical University of Silesia, Sosnowiec, Katowice, Poland; 8 Department of Radiology, University Hospital Basel, Basel, Switzerland; University of Pécs Medical School, HUNGARY

## Abstract

**Objective:**

Here, we retrospectively investigate the value of voxel-wisely plotted diffusion tensor-derived (DTI) axial, radial and mean diffusivity for the early detection of malignant transformation (MT) in WHO II glioma compared to contrast-enhanced images.

**Materials and Methods:**

Forty-seven patients underwent brain magnetic resonance imaging follow-up between 2006–2014 after gross-tumor resection of intra-axial WHO II glioma. Axial/Mean/Radial diffusivity maps (AD/MD/RD) were generated from DTI data. AD_min_/MD_min_/RD_min_ values were quantified within tumor regions-of-interest generated by two independent readers including tumor contrast-to-noise (CNR). Sensitivity/specificity and area-under-the-curve (AUC) were calculated using receiver-operating-characteristic analysis. Inter-reader agreement was assessed (Cohen’s kappa).

**Results:**

Eighteen patients demonstrated malignant transformation (MT) confirmed in 8/18 by histopathology and in 10/18 through imaging follow-up. Twelve of 18 patients (66.6%) with MT showed diffusion restriction timely coincidental with contrast-enhancement (CE). In the remaining six patients (33.3%), the diffusion restriction preceded the CE. The mean gain in detection time using DTI was (0.8±0.5 years, p = 0.028). Compared to MD_min_ and RD_min_, ROC-analysis showed best diagnostic value for AD_min_ (sensitivity/specificity 94.94%/89.7%, AUC 0.96; p<0.0001) to detect MT. CNR was highest for AD (1.83±0.14), compared to MD (1.31±0.19; p<0.003) and RD (0.90±0.23; p<0.0001). Cohen’s Kappa was 0.77 for AD_min_, 0.71 for MD_min_ and 0.65 for RD_min_ (p<0.0001, respectively).

**Conclusion:**

MT is detectable at the same time point or earlier compared to T1w-CE by diffusion restriction in diffusion-tensor-derived maps. AD demonstrated highest sensitivity/specificity/tumor-contrast compared to radial or mean diffusivity (= apparent diffusion coefficient) to detect MT.

## Introduction

Supratentorial astrocytoma, oligoastrocytoma and oligodendroglioma are WHO grade II tumors and represent a biologically heterogeneous group, with an incidence of 1-2/100.000 per year [1]. These low-grade gliomas (LGGs) have low mitotic activity and lack histological aggressive features. However, they have a high propensity for recurrence after attempted resection due to diffuse infiltration patterns into surrounding tissue resulting in high probability for subtotal resection. LGGs typically progress to high grade gliomas due to slow accumulation of somatic mutations over time [2,3] known as malignant transformation (MT) into glioma WHO grade III and after additional mutations to secondary glioblastoma WHO grade IV which has a dismal prognosis, resulting in rapidly progressive decline and death in almost all patients [4]. MT is marked by a rapid progression of the tumor size, cellularity and angiogenesis [2] and occurs on average between 2.1 to 10.1 years after diagnosis [5], depending on previously obtained treatment such as radiotherapy [6]. The high variability in the duration of the progression-free interval impedes the prediction of the exact time point of histopathological progression and therefore necessitates regular and relatively frequent imaging follow-up to detect MT as soon as possible. New appearance of contrast enhancement (CE) in magnetic resonance imaging (MRI) follow-up is currently the established imaging feature related to MT [7,8], especially if no recent therapy has been administered. CE reflects contrast extravasation from tumor neovasculature resulting from breakdown of the blood-brain barrier (patchy and faint CE pattern) and stems partly from the effects of intravascular Gadolinium in tumor vessels (nodular CE pattern) [4,9,10]. However, CE is known to have limitations as a tool for tumor characterization: (i) up to 30–50% of grade I or II gliomas may display CE in the absence of MT [11], (ii) 30% gliomas may lack CE despite MT [12,13], (iii) oligodendrogliomas are known for their avid and heterogenous CE which impedes accurate staging based on CE alone [14], (iv) surgical resection or radio-chemotherapy may itself cause CE [15], hampering the distinction between pseudo-progression and true progression especially in the early post-treatment interval [14].

Diffusion-weighted imaging, which probes tissue microstructure, is used as a surrogate marker for the increased cellularity resulting from elevated mitotic activity in higher-grade brain tumors [16–19]. From a histological perspective, cellularity may indicate MT without neoangiogenesis being present [2] explaining why a significant proportion of high-grade gliomas do not necessarily need to show CE [20]. In the case of glioblastoma, early changes in diffusion imaging prior to CE have been demonstrated [21]. In this longitudinal retrospective study on WHO II glioma patients, we hypothesize that increased cellularity, assessed using diffusion-tensor-imaging(DTI)-derived maps, precedes CE and thus potentially facilitates an early prediction of transformation into anaplastic WHO III glioma.

## Methods

### Participants

This retrospective single-institution study was approved by the local ethics committee (Bioethics Committee of Medical University of Silesia in Katowice) and conducted in agreement with the declaration of Helsinki. Due to the retrospective nature of the study, written informed consent was waived. Patient data was anonymized prior to analysis. Sixty-eight patients with a diagnosis of an intra-axial supratentorial WHO II brain tumor (astrocytoma, oligoastrocytoma, oligodendroglioma) were under MR surveillance after surgical resection between January 2006 and January 2014 at the Maria Sklodowska-Curie Memorial Cancer Centre, Gliwice, Poland. The primary diagnosis was confirmed by histopathology in all patients using tissue samples from either resection or biopsy. Inclusion criteria were (i) WHO II low-grade tumor with gross-tumor resection, (ii) follow-up over at least 2 years. Patients were excluded if 1) DTI data was missing, incomplete or unreadable due to artifacts, 2) prominent post-surgical bleeding was present thus leading to false-low diffusivity values. A patient was regarded as progressive if either one of the two criteria applied: 1) histopathological confirmation by re-biopsy or re-operation or 2) radiological confirmation defined as the combination of a new CE in a previously non-enhancing area and subsequent rapid expansion of the tumor in at least two subsequent follow-ups.

### Imaging

MRI (Table 1) was performed on a Siemens Avanto 1.5 Tesla (Siemens, Erlangen, Germany). b-Values for DTI were 0, 1000 s/mm^2^ and 12 diffusion directions were used. DTI data was post-processed using MITK Diffusion [22] to generate voxel-wisely calculated maps of axial, mean and radial diffusivity (AD, MD and RD), which were derived from the eigenvalues λ_1_, λ_2_ and λ_3_ of the diffusion tensor:

$$AD=\lambda_{1};RD=\frac{{(\lambda }_{2}+\lambda_{3})}{2};MD=\frac{{(\lambda_{1}+\lambda }_{2}+\lambda_{3})}{3};\;{\lambda_{1}\geq \lambda }_{2}\geq \lambda_{3}$$

**Table 1:** MRI protocol between 2006–2014. For T1w-CE, patients were injected with 0.1mmol/kg of Gadobutrol (Gadovist, Bayer Healthcare, Germany). b-values for DTI were 0, 1000 s/mm^2^ for 12 diffusion directions.

Sequence	Resolution mm^3^	FOV mm^2^	Matrix	TE/TR ms	slices	Time of acquisition
T1w native SE	0.6 x 0.6 x 5	250 x 188	448 x 336	16/575	21	3 min 14 sec
T1w GRE CE (MPR)	1 x 1 x 1	250 x 250	256 x 256	4.1/1080	176	4 min 36 sec
T1w-CE SE	0.6 x 0.6 x 5	250 x 188	448 x 336	16/470	21	5 min 17 sec
DTI	1.8 x 1.8 x 4	230 x 230	128 x 128	104/7200	25	12 min 09 sec
T2w-TIRM	0.7 x 0.7 x 5	250 x 188	384 x 288	88/8000	21	4 min 32 sec

FOV: field of view. GRE: gradient echo; CE: contrast-enhanced; DTI: diffusion tensor imaging; MPR: multi-planar reconstruction; SE: spin-echo; TIRM: turbo-inversion recovery magnitude

### Region of interests (ROIs)

**Fig 1:**
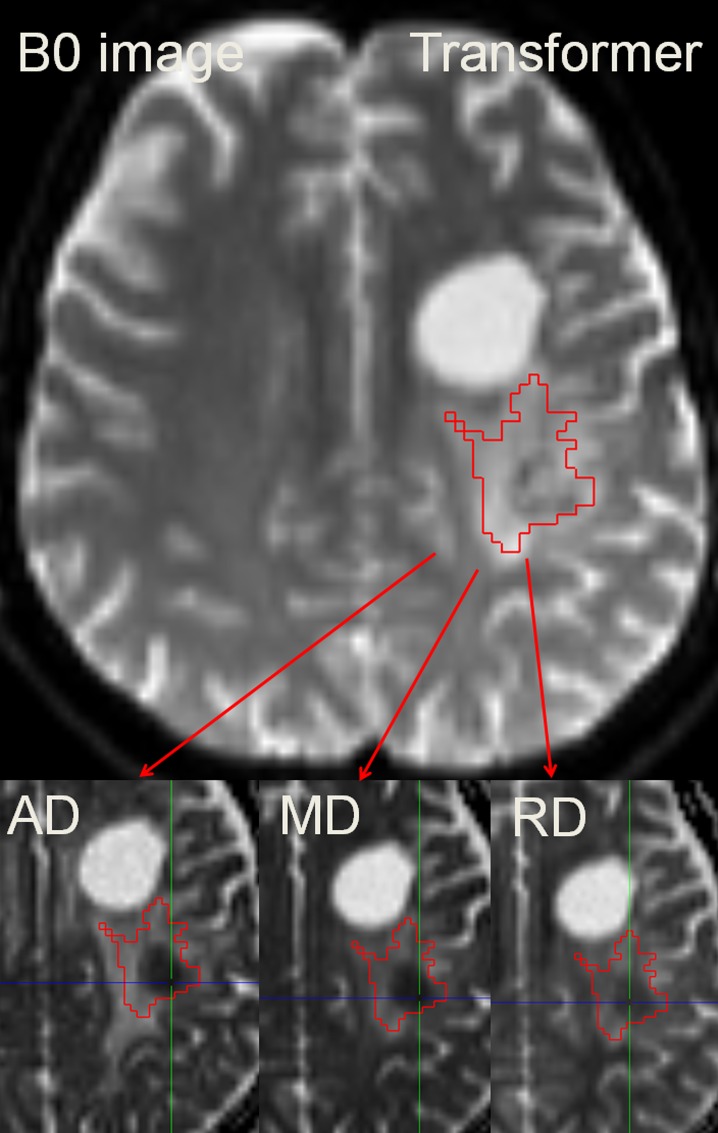
Segmentation of the T2w-hyperintensity zone to derive AD_min_/MD_min_/RD_min_. Because DTI-maps are intrinsically co-registered the ROI may easily be transferred to each of the employed parameter maps. The colored crosshair in the images indicates the localization of the automatically determined minimum value. In the given example, the patient was diagnosed with anaplastic transformation.

All ROIs were created by two readers (M.T.F. with 5 years of experience, B.S. with 12 years of experience in oncologic MRI of the brain) using MITK Diffusion. Both readers placed the ROIs independently and were blinded to the histopathology that was obtained by B.B.B., H.M. and R.T. In the group with MT, the exam date at which CE was first detected was used for tumor segmentation. A 2D-ROI was drawn on the non-diffusion weighted b_0_-map defining the complete T2w-hyperintensity that was always visible adjacent to the resection cavity (Fig 1). Additionally, tumor-ROIs and ROIs defining the T2w-hyperintense brain were created for contrast-to-noise (CNR) measurements. In patients who did not experience an occurrence of CE during the study period, segmentation was based on the latest available examination date in the T2w-hyperintense brain tissue.

CNR of the tumor and the surrounding T2w-hyperintense tissue was calculated as follows:

$$CNR=\frac{\frac{D_{T2w}(reader1)+D_{T2w}(reader2)}{2}-\frac{D_{tumor}(reader1)+D_{tumor}(reader2)}{2}}{STD}$$

D represents the average AD, MD, or RD values of the T2w-hyperintensity area (T2w) or of the tumor ROI (tumor) per reader. The standard deviation STD is calculated as given:

$$STD=\frac{{DSTD}_{T2w}(reader1)+{DSTD}_{T2w}(reader2)}{2}+\frac{{DSTD}_{tumor}(reader1)+{DSTD}_{tumor}(reader2)}{2}$$

DSTD represents the standard deviation of the T2w-hyperintensity area (T2w) or of the tumor ROI (tumor) per reader calculated for AD, MD and RD maps.

### Qualitative image analysis

For qualitative evaluation, focal clusters of low intensity on DTI-derived parametric maps within the tumor ROI were noted as suspicious for MT. Subsequently, T_1_-weighted post-contrast imaging was evaluated and the time differences between the occurrence of transformation (focal restriction in DTI, contrast-enhancement in T1w-CE) in both exams was noted. Furthermore, we descriptively evaluated the spatial concordance/difference between the DTI-derived maps and conventional imaging and the temporal evolution of the identified regions with diffusion restriction.

### Quantitative Image analysis

A quantitative data analysis was performed to determine the optimal DTI parameter for detection of MT. As DTI-derived parameter maps are mathematically calculated from the DTI raw data, no additional registration steps are needed and the same ROI can be used for parametric calculations on all maps (AD/MD/RD). Mean and minimum parametric measurements within each tumor-ROI were calculated automatically using MITK Diffusion. Automatic ROI-readout was performed using a custom-built tool coded in Visual-C# language. CNR was calculated by placing two ROIs (tumor, surrounding T2w edema). A rigid registration was used to co-register DTI datasets to T2w-FLAIR and T1w-CE to assess the spatial relationship between DTI parametric changes and CE. In patients in whom diffusion restrictions were noticed earlier than contrast-enhancement, a ROI was placed in the area of the T2w hyperintensity and compared to the stable patients.

### Statistical analysis

Statistical analysis was performed with SPSS 20 (IBM, Armonk, New York, USA). For all tests, a p-value < 0.05 indicated statistical significance. Initially, the normal distribution of data was evaluated using a Shapiro-Wilk test. Variance homogeneity of Ad_min_/MD_min_/RD_min_ was tested using Levene’s test. To compare the time point of transformation based on CE and DTI and for the comparison of CNR measurements, the Wilcoxon Signed-Rank test for paired samples was used. Inter-group differences in data distribution were visualized using box-plots and tested for significance by using univariate analysis of variance (ANOVA) including Bonferroni-correction for multiple hypothesis testing. Bonferroni correction was used to correct for multiple comparisons. To calculate sensitivity and specificity at time point of contrast-enhancement, a receiver-operating-characteristics (ROC) analysis was performed in which the three parameters of each patient and the status of transformation (0 = no, 1 = yes), indicated by the transformation criteria, was assigned per patient. The positive predictive value was calculated for AD_min_/MD_min_/RD_min_, respectively. Inter-reader agreement between both readers was calculated using Cohen’s kappa for the ROIs comparing diffusion restrictions and contrast-enhancement at the same time point. The agreement of both readers was quantified using a weighted kappa-analysis (0<*κ*≤0.20 slight, 0.20<*κ*≤0.40 fair, 0.40<*κ*≤0.60 moderate, 0.60<*κ*≤0.80 very good and 0.80<*κ*≤1 substantial agreement).

## Results

### Patients

**Fig 2:**
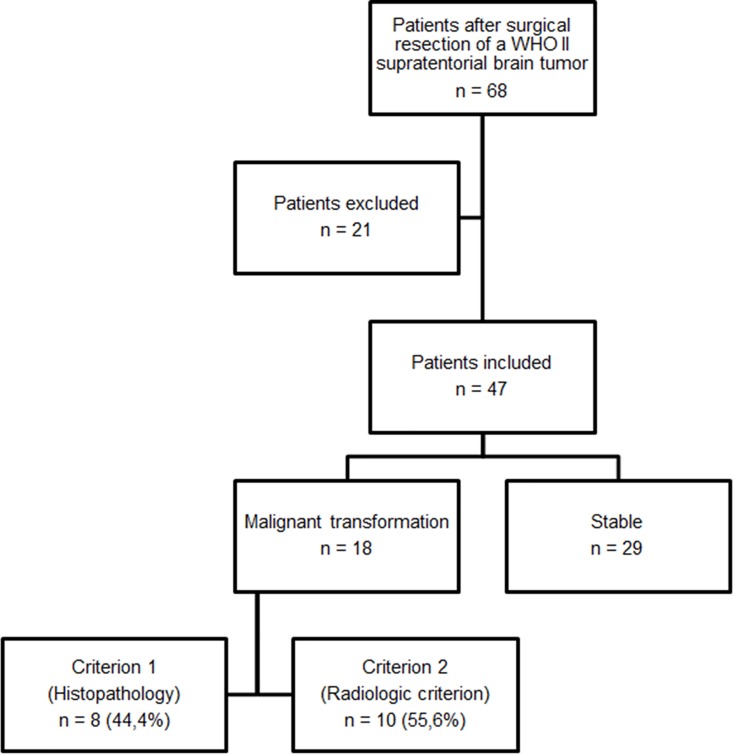
Flow-chart of included patients.

Of the inital 68 patients, 21 patients were excluded (Table 2, Fig 2). Of the remaining 47 patients (19 female [37.7 ± 9.2 years], 28 male [37.5 ± 11.1 years]), 29 had no transformation (7 female [41.3 ± 9.9 years], 22 male [38.3 ± 12.0 years]) and 18 showed MT (12 female [35.7 ± 8.5 years], 6 male [34.8 ± 6.8 years]). In these 18 patients, transformation was histopathologically confirmed in eight and radiologically in ten. Of 47 included patients, mean number of follow-up MRI examinations was (8.3 ± 3.1) per patient.

**Table 2:** Tumor histology of finally included patients determined after surgical resection according to the WHO 2007 criteria[2].

Histopathological diagnosis	Malignant transformation	Stable	Sum
Astrocytoma WHO II	13	16	29
Astr. protoplasmaticum	1	3	(4)
Astr. fibrillare	9	10	(19)
Astr. gemistocyticum	3	3	(6)
Oligoastrocytoma WHO II	3	11	14
Oligodendroglioma WHO II	2	2	4
Sum	18	29	47

### Qualitative analysis with regard to obtained therapy

**Fig 3:**
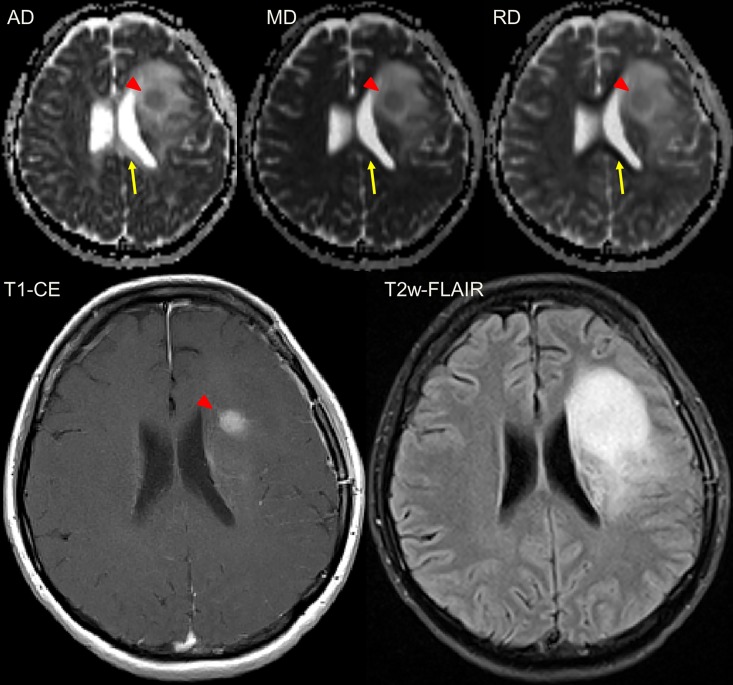
Female patient (43y) with a malignant transformation of a primary gemistocytic Astrocytoma WHO II. The transformation (red triangle) is visible in the T1w-CE images and at the same timepoint also in the diffusion tensor-derived parameter maps axial (AD), mean (MD) and radial diffusivity (RD). The area of changed brain tissue is most extensive in the T2w-FLAIR. Please note that only in the axial diffusivity map, white matter is depicted hyperintense (yellow arrow) which leads to difference in contrast. The T2 hyperintense region in the T2 FLAIR is matching with the hyperintense region in the DTI-parameter maps.

**Fig 4:**
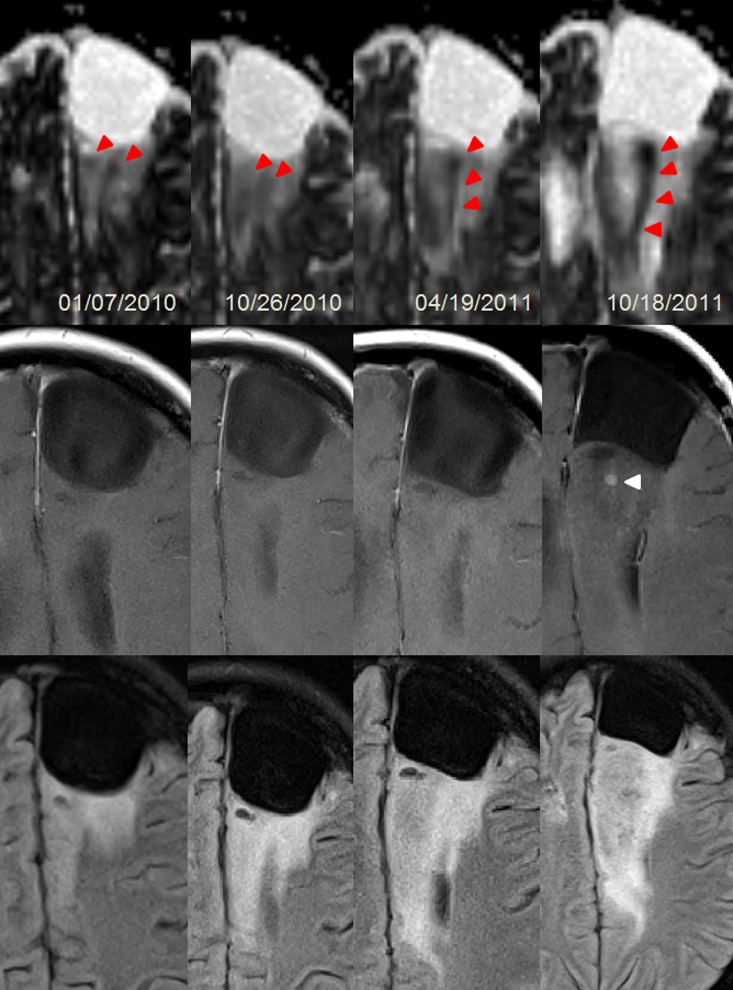
Female patient (24y) with a malignant transformation (anaplastic astrocytoma WHO III) of a primary fibrillary Astrocytoma WHO II. The transformation according to currently accepted radiologic definition was visible as a new punctual CE at the last column. However, changes in cellularity (red triangles) were preceding CE (white triangle) more than one year. The area of the most focal diffusion restriction corresponds to the focal uptake. Upper row: Axial diffusivity maps; Middle row: T1w-CE; Bottom row: T2w-FLAIR. Dates are given in day/month/year.

**Fig 5:**
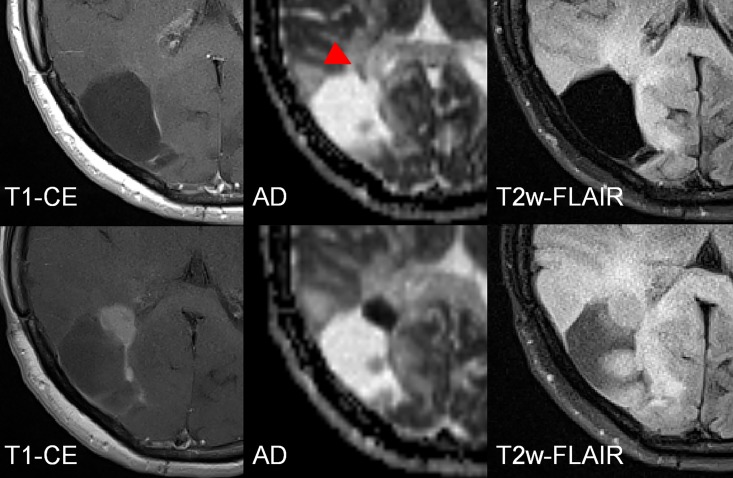
Female patient (41y) with a malignant transformation (anaplastic astrocytoma WHO III) of a primary resected fibrillary Astrocytoma WHO II in the right occipital lobe. In the first examination (upper line) a subtle diffusion restriction is observed (red triangles) that predicts the future growth (lower line) of the malignant transformation, indicated by a hypointense cluster in the AD map. The difference between the first examination and the follow-up is 5 ½ months.

**Fig 6:**
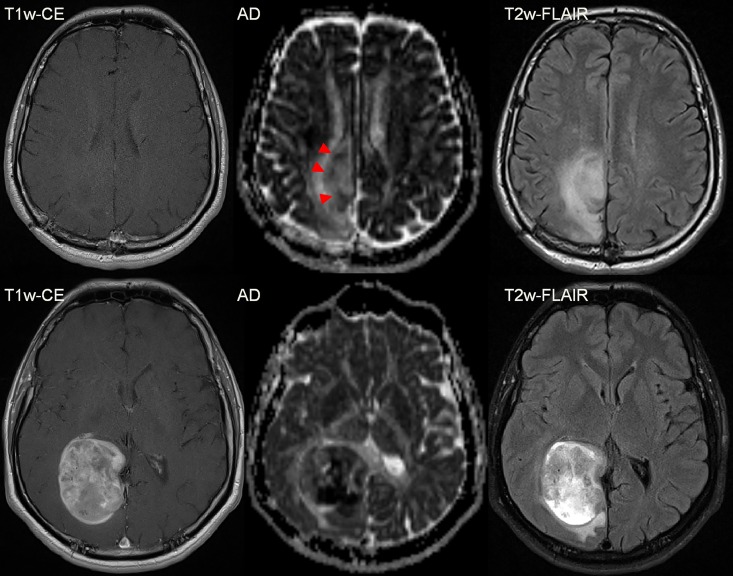
Male patient (42y) with a malignant transformation (anaplastic astrocytoma WHO III) of a primary resected fibrillary Astrocytoma WHO II in the right occipital lobe (resection cavity is not shown). Focal, small diffusion restrictions predict the anaplastic transformation before CE appears. The difference between the first examination (upper row) and the follow-up (bottom row) is 20 months.

In all 18 patients with MT, clustered diffusion restrictions were visible in the T2w-hyperintensity zone, the latter matching with hyperintensity in DTI-derived maps AD, MD and RD (Fig 3). Especially in the typical T2w-FLAIR hyperintense region that is also depicted hyperintense in the AD/MD/RD maps, the recurrence is well contrasted and can be readily detected (Figs 4–6). In the evolution of malignization (Fig 4), the same growth pattern in the DTI-derived maps was observed in all patients: (i) Resection of the glioma leads to a cavity filled with cerebrospinal fluid (ii) adjacent to the resection cavity, a T2w-hyperintense zone evolves or may be present already directly after the surgery, potentially harboring residual tumor cells in both controls and transformers (iii) in patients with transformation within this T2w-hyperintense zone, the recurrence evolves with increasing diffusion restriction over time.

Considering the qualitative comparison of DTI-derived parameter maps compared to CE, 12 of the 18 patients (66.6%) with recurrence showed a clear diffusion restriction coincidental with the CE (as in Fig 3). In the remaining six patients (33.3%), the diffusion restriction preluded the CE (as in Figs 4–6). The mean gain in detection time using DTI was (0.8 ± 0.5 years) and was statistically significant (p = 0.028). None of the 6 patients in whom DTI detected the transformation earlier than CE received adjuvant radiotherapy, five of them were diagnosed with anaplastic astrocytoma and one with anaplastic oligo-astrocytoma after transformation. Of the remaining 12 transformers, 8 received adjuvant radiotherapy 1–3 months post-operation. In total, 38 patients were undergoing adjuvant radiotherapy of which 6 patients were receiving multiple sessions. There was no adjuvant chemotherapy applied. After MT was diagnosed, 7 of 18 patients received progression-related radiation therapy.

**Fig 7:**
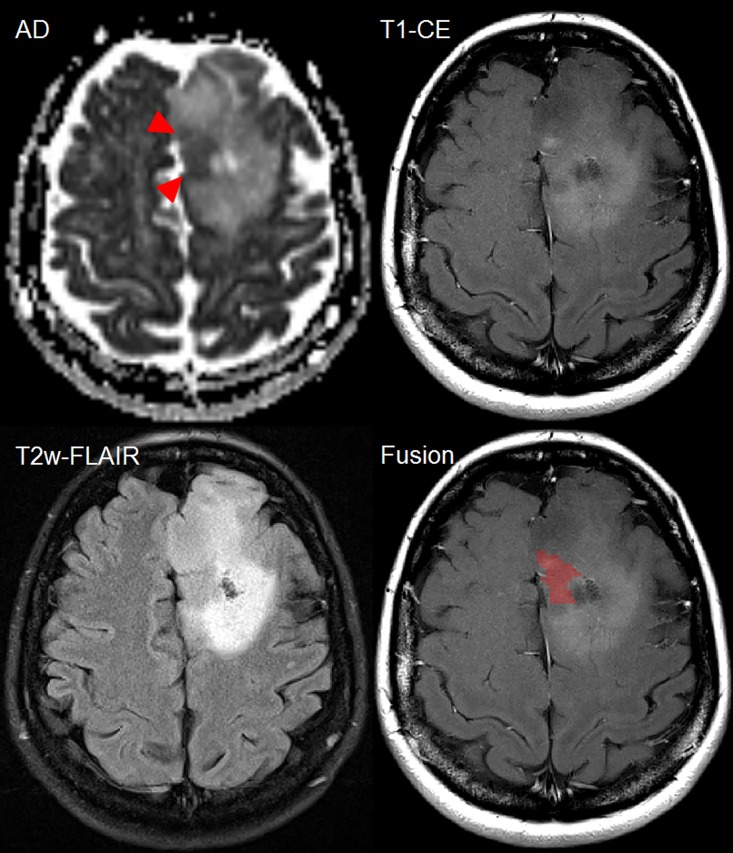
Female patient (44y) with malignant transformation of a primary resected oligoastrocytoma WHO II into anaplastic oligoastrocytoma III in the left frontal lobe demonstrating heterogeneity within the patchy contrast-enhancing region. Increased diffusion restriction (red triangles) is noted at the same timepoint as new CE. Within the patchy area of CE, the spatial position of hypercellularity is visualized as red cluster (last image). Comparing T2w-FLAIR and axial diffusivity, the hypercellularity is found to be located in a T2w-hypointense region with a punctual CE focus. However, large proportions of patchy CE do not match with the diffusion restriction. Such information is important to consider for guidance of stereotactic biopsy and focal treatment planning.

In 11 of 18 (61.1%) patients with MT, spatial distribution of CE and DTI were concordant (as in Fig 3). Spatial mismatch between DTI and CE was observed in 7 of 18 (38.9%) patients with MT (as in Fig 7).

### Quantitative image analysis

**Fig 8:**
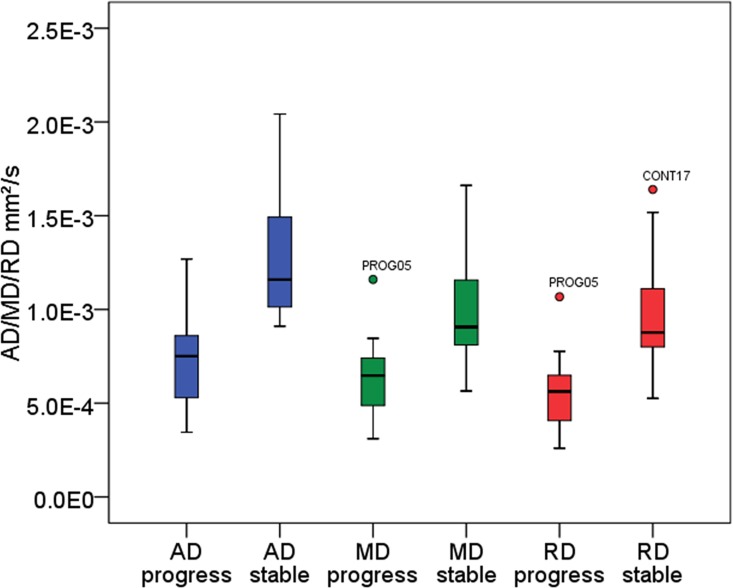
Boxplot analysis of obtained ROI-averaged diffusivity parameters to visualize group differences (stable versus progress). Plotted values were obtained at the first appearance of CE (progress) or at the last acquired timepoint available (stable). Quantitative results including p-values are given in Table 3.

**Fig 9:**
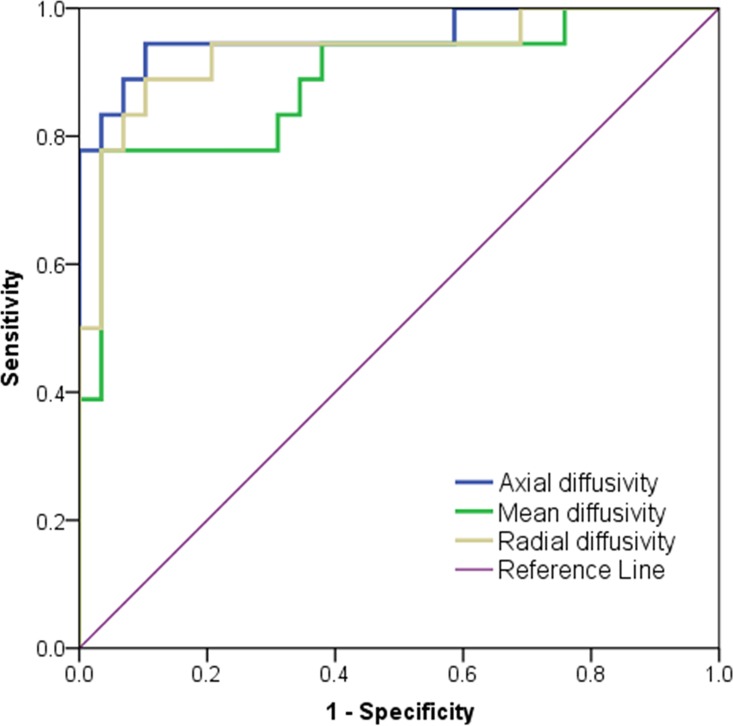
Reciever operating characteristic analysis of minimum diffusion tensor parameters. Quantitative results are given in Table 4.

Normal distribution and variance homogeneity was confirmed for AD, MD and RD values. In the inter-group comparison, mean and minimum values of AD, RD and MD were significantly lower in transformers compared to non-transformers at the date of CE (p<0.0001, respectively) (Fig 8, Table 3). ROC-analysis showed best diagnostic value among the three minimum-parameters for AD_min_ (sensitivity 94.94%, specificity 89.7%, area under the curve 0.96) (Fig 9, Table 4). Positive predictive values of AD_min_/MD_min_/RD_min_ for the given cut-offs in Table 4 were 81.0%/60.7%/73.9%. In the 18 patients with transformation, tumor-CNR was significantly highest for AD (1.83±0.14), compared to MD (1.31±0.19; p<0.003) and RD maps (0.90±0.23; p<0.0001). In 6 patients of the MT group mean AD_min_/MD_min_/RD_min_ values were 0.82±0.09/0.73±0.05/0.62±0.07 mm^2^/s at a timepoint in which new CE indicative for MT lacked at the same timepoint but appeared in later follow-ups. This signal drop of 64.1%/73.0%/63.9%, resulting from the formation of diffusion restrictions compared to the homogeneous T2w hyperintensity region of stable patients indicated early transformation. Cohen’s Kappa for inter-reader agreement was 0.77 for AD_min_, 0.71 for MD_min_ and 0.65 for RD_min_ (p<0.0001, respectively) in patients with transformation (n = 18) indicating a very good agreement between the two readers.

**Table 3:** Quantitative analysis of group-wise data distribution regarding AD_min_/MD_min_/RD_min_ as visualized in Fig 8, given for 18 patients with MT versus 29 stable patients at the same time point of follow-up. In 6 patients of the MT group mean AD_min_/MD_min_/RD_min_ values were 0.82±0.09/0.73±0.05/0.62±0.07 mm^2^/s with a signal drop of 64.1%/73.0%/63.9% compared to the stable group in the examination prior to first CE appearance thus indicating early MT.

Parameter	Group	(Mean ± Std. Dev.) × 10^−3^ mm^2^/s	[95% confidence interval] × 10^−3^ mm^2^/s	p-value (ANOVA)	F (ANOVA)
Axial diffusivity	Stable	(1.28 ± 0.31)	[1.16–1.4]	p < 0.0001*	41.614
	Malignant transformation	(0.72 ± 0.23)	[0.61–0.84]		
Mean diffusivity	Stable	(1.00 ± 0.26)	[0.90–1.1]	p < 0.0001*	24.446
	Malignant transformation	(0.64 ± 0.21)	[0.53–0.74]		
Radial diffusivity	Stable	(0.97 ± 0.26)	[0.87–1.07]	p < 0.0001*	33.023
	Malignant transformation	(0.56 ± 0.20)	[0.46–0.66]		

*Bonferroni corrected.

**Table 4:** Reciever operating characteristic analysis of minimum values (averaged between the two readers). The test was positive for recurrence if the variable results were less than or equal to the cut-off value. Axial diffusivity was found to have superior diagnostic value (sensitivity, specificity, area under the curve and positive predictive values) to detect the recurrence compared to mean and radial diffusivity. Positive predictive values of AD_min_/MD_min_/RD_min_ for the given cut-offs were 81.0%/60.7%/73.9%.

Parameter	Cut-off value × 10^−3^ mm^2^/s (Sensitivity/Specificity)	AUC*	Std. Error	Asymptotic significance	[95% confidence interval] × 10^−3^ mm^2^/s
Axial diffusivity	0.96 (94.4%/89.7%)	0.96	0.34	p < 0.0001	[0.89–1.00]
Mean diffusivity	0.85 (94.4%/62.1%)	0.89	0.53	p < 0.0001	[0.78–0.99]
Radial diffusivity	0.79 (94.4%/79.3%)	0.93	0.42	p < 0.0001	[0.85–1.00]

*AUC: area under the curve

## Discussion

The event of MT of a LGG into its anaplastic WHO III equivalent is timely unpredictable but nearly inevitable and may occur even after decades. This explains why imaging-based studies on predicting MT in the present literature are usually limited to small patient numbers [23–25] and why existing data on this topic is scarce.

The gradual mutations evolving in WHO II glioma lead to two mainstays of MT: an increase in cellularity followed by neo-angiogenesis [2,3]. The latter is depicted using contrast-enhanced T1w MRI and dynamic perfusion imaging and is currently the standard for diagnosing MT [8]. The second may be visualized using diffusion-weighted imaging (DWI), usually assessed using high b-value images and derived apparent-diffusion-coefficient (ADC)[26,27]. Due to diffusion restriction caused by high cellularity, the ADC in these areas is reduced [17]. In the present study, DTI as a special form of DWI also measuring the directionally dependent diffusion was used and DTI-derived AD/MD/RD maps were radiologically evaluated for potential occurrence of new diffusion restrictions indicating MT including time-associated appearance and spatial distribution compared to CE. We used AD_min_, MD_min_ and RD_min_, where MD is equivalent to the ADC. A meta-analysis comprising 729 patients provided evidence that a low ADC significantly correlates with a high cellularity in the brain [28]. It was demonstrated that ADC_min_ is significantly associated with cellularity and prognosis in WHO III and IV brain tumors [29–31] and may be used for grading between them [32]. An advantage of minimum values is that they are easily quantifiable and robust to investigator-dependent differences in ROI placement substantiated by the very good agreement between both readers. While the MD itself is mathematically representing the average signal decay with increasing b-value, the scalar indices AD and RD are able to differentiate effects along or perpendicular to the main diffusion direction. Previously, the clinical relevance of AD and RD [33] has not been investigated extensively in brain tumors and parametric maps of the scalar indices were not aqcuired.

Our results revealed highest tumor-CNR, highest combined sensitivity/specificity/AUC and the highest positive predictive value for AD_min_ compared to MD_min_/RD_min_ indicating that the most prominent effect in the signal depicting MT stems from a diffusion restriction along the main diffusion direction. Higher CNR is explained by hyperintensity of white matter in AD in contrast to MD/RD. However, a direct microstructural interpretation of the AD and RD quantitative values in relation e.g. to myelinisation or axonal degeneration is highly speculative [34] and thus avoided here. However, our findings do indicate that it may be advantageous to switch from the commonly used DWI protocol that only allows for the extraction of the ADC/MD, to a DTI protocol that also yields the AD. With current state of the art scanners, this can be realized without significantly increasing the measurement time.

The second finding of interest of the present study was that diffusion restrictions could be observed prior to CE in 6/18 patients with MT, while 12/18 demonstrated CE at the same time. The 6/18 patients did not receive adjuvant radiotherapy after surgical resection. Therefore, a therapy effect of patients with MT undergoing adjuvant radiotherapy (8/18) could have suppressed cell growth in some patients resulting in later appearance of diffusion restrictions together with CE. Hypercellularities preceding CE were biologically meaningful as they predicted the future region of focal uptake in all 6 cases. Here, no spatial mismatch between CE and DTI was noticed. In agreement with this finding, early patterns of high-grade glioma infiltration using DWI and DTI were reported [21,35,36], however, in these studies the scalar indices were not visualized. Our findings are further substantiated by the fact that both MR-spectroscopy [23,25] and perfusion MRI [24,37] may detect abnormalities before CE appears. In conclusion, AD may depict the correlate for these early metabolic and micro-vascular changes. The decrease of AD (cut-off 0.96 mm^2^/s) indicates MT underlining that increase in cellularity within the T2w-hyperintense region has fundamental consequences for the patient and his prognosis. An open question that warrants further investigation is if DTI can shorten the time to detection of MT when the examination interval itself is shortened. Since the DTI exam alone is very short (max. 5 minutes) and requires no contrast agent, it could be considered to interleave the current follow-up intervals with a short DTI-only exam to evaluate this.

The partial lack of spatial correlation of the observed hypercellular clusters within T1w-CE or T2w-FLAIR images illustrates that focal diffusion restrictions do not need to appear in the complete area of CE (Fig 7). In 38.9%, the spatial distribution of maximum CE and maximum diffusion restriction was different indicating that patchy leakage of contrast agent may be misleading to indicate the area of MT. Hypercellular clusters likely reflect the typical heterogeneity observed in high-grade glioma [38] indicating that some areas reveal a significant higher cell density. This finding may explain histopathological sampling errors [11], reported especially to occur in tumors with low proliferative activity and mixed gliomas [39]. For biopsy targets, usually the area of the most prominent CE is taken but if the CE is patchy, then the area of highest diffusion restriction may be missed. Mapping these clusters (Fig 7) on T1w-CE images demonstrates promising potential for a better targeting of high-cellular structures. Previous studies have compared histology to diffusion markers and significant correlation between high-grade glioma and diffusion markers has been demonstrated [31,40]. Future studies should assess the value of using pre-biopsy DTI to target the highest area of diffusion restriction if suspicion for MT is raised.

The present study has limitations including biases resulting from the retrospective design of the present study. Furthermore, only 8/18 patients underwent a second biopsy so the diagnosis for MT needed to be undertaken by using a commonly accepted radiological criterion [8] where a rapidly size-changing tumor combined with new CE in a previously non-enhancing area is assumed to be transformed, confirmed by at least two follow-ups in order to exclude pseudo-progression. This serves as predictor of the patient’s course and is an accepted radiologic evidence of MT in those cases where a biopsy cannot be obtained [8]. Furthermore, as mentioned earlier, a negative biopsy does not exclude MT and the general limitation of false-negative histopathology assessed by biopsy due to sampling errors is known. Thirdly, we carefully screened the data for any bias which could potentially negatively influence the automatic data acquisition (minimum value). It was checked by both readers whether the automatically determined minimum value is located in a voxel incorporating artifacts resulting from bleeding or susceptibility. However, we cannot completely rule out such biases because histological evidence of transformation could not be obtained in 10/18 patients. In the present study, only 4 oligodendrogliomas were included in total. Therefore, the findings of the present study are mainly representative for astrocytoma and oligoastrocytoma. At last, we did not co-evaluate other advanced quantitative imaging methods, e.g. perfusion imaging.

Summarized, DTI-derived metrics provide valuable information on MT. The present study provides evidence that the parallel screening for focal diffusion restrictions in AD maps is useful to detect MT of resected LGG, particularly because DTI is easily acquirable and not dependable on contrast media. The reliability of DTI is limited directly after surgery by deposition of blood products. However, decrease of AD into hypointense focal clusters, not relatable to artificial decrease by hemorrhage or calcifications, indicates MT and should be recognized. Increase in cellularity is a histological feature of MT [2] and is depictable using DTI. Therefore, decrease of AD (below 0.96 mm^2^/s) provides an additional parameter that may be used to substantiate or raise suspicion for MT. Furthermore, the present study recommends assessment of AD maps in general, since the intrinsic contrast of tumor to surrounding tissue was found to be slightly higher compared to the MD map (= ADC).

For future studies, it would be highly interesting to assess its implementation into a multi-parametrical setup in combination with positron emission tomography (PET/MRI) to evaluate which of the current methods available—including spectroscopy, DTI and perfusion MRI—achieves highest diagnostic accuracy to detect MT at the earliest possible time point.

## Conclusion

The present study provides evidence that the parallel screening for focal diffusion restrictions with low axial diffusivity values within the corresponding area of T2w-hyperintensity is valuable for patients under surveillance of gross-tumor resected WHO II gliomas. Axial diffusivity demonstrated highest sensitivity/specificity/tumor-contrast compared to radial and mean diffusivity (= apparent diffusion coefficient) to indicate malignant transformation. In a subgroup of patients, early changes in axial/mean/radial diffusivity maps may be noticed even before conventional contrast enhancement appears and signalize early malignant transformation.

## Supporting Information

S1 File This statistics file contains the quantitative data. (SAV)

## Abbreviations

AD/MD/RD: axial, mean and radial diffusivity
ADC: apparent diffusion coefficient
CE: contrast enhancement
CNR: contrast-to-noise ratio
DTI: diffusion tensor imaging
DWI: diffusion-weighted imaging
FOV: field of view
LGG: low-grade glioma
MRI: magnetic resonance imaging
MT: malignant transformation
ROC: receiver operating characteristics analysis
ROI: region of interest
